# Supplementary material for: Local orchestration of distributed functional patterns supporting loss and restoration of consciousness in the primate brain
Source: Nat Commun. 2024 Mar 11;15:2171. doi: 10.1038/s41467-024-46382-w (PMC10925605; doi:10.1038/s41467-024-46382-w)
Supplement: Supplementary file 1 — Supplementary Information [file 41467_2024_46382_MOESM1_ESM.pdf]

Local Orchestration of Distributed Functional Patterns Supporting Loss and Restoration of Consciousness in the Primate Brain

Luppi, Uhrig, Tasserie *et al.*

Supplementary Information

Supplementary Figures

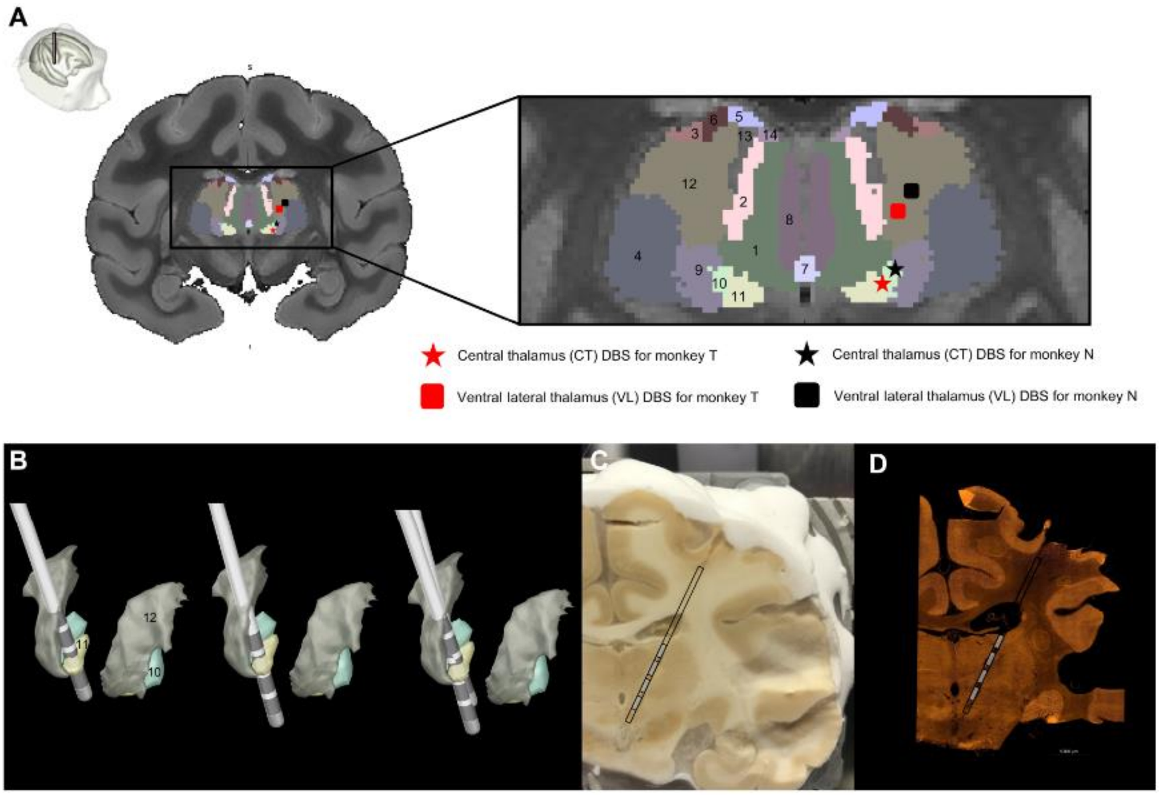

Figure S1. Electrode locations for Deep Brain Stimulation of the centro-median and ventral lateral thalamus of the macaque. (A) Anatomical localization of the DBS lead and active contacts. Coronal section of an anatomical MRI. Zoomed-in view of the thalamic nuclei as segmented with the CIVM atlas. DBS was delivered to either the CT (star label) or the ventral lateral thalamus (VL) (square label). 1, mediodorsal nucleus, central part; 2, mediodorsal nucleus, lateral part; 3, ventral lateral nucleus, lateral part; 4, ventral posterolateral nucleus; 5, lateral dorsal nucleus, superficial part; 6, ventral anterior nucleus, lateral part; 7, intermediodorsal nucleus; 8, mediodorsal nucleus, medial part; 9, ventral posteromedial nucleus; 10, CM nucleus, lateral part; 11, CM nucleus, medial part; 12, ventral lateral nucleus, medial part; 13, centrolateral nucleus; 14, mediodorsal nucleus, dorsal part. (B) Automated electrode reconstruction and three-dimensional (3D) rendering in monkey T (left), in monkey N (center), and in both monkeys (right). (C and D) Coronal histological section, corresponding to the level interaural 8.40 mm. Both cryostat image (C) and NeuN immunohistochemistry image (D) confirm the positioning of the active contacts within thalamic nuclei. Adapted from Figure 2 of [1], originally published under CC-BY license.

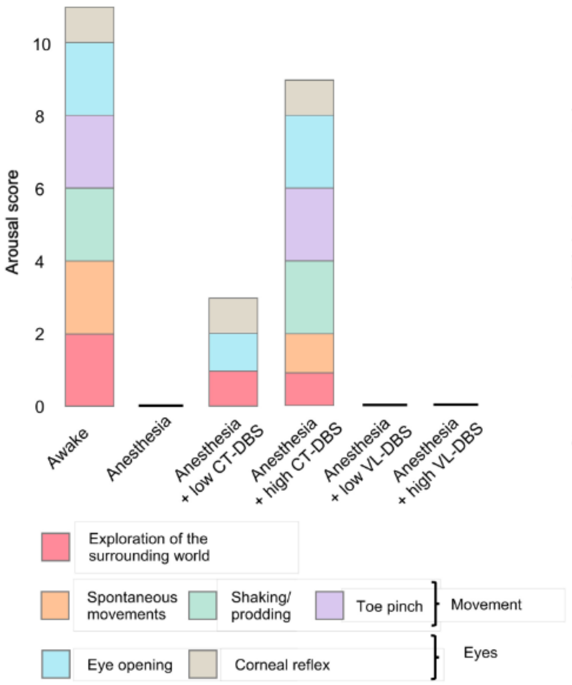

Figure S2. Effects of thalamic DBS on arousal in anaesthetized monkeys, as a function of the electrode location and the level of stimulation (low-amplitude versus high-amplitude DBS). Only the stimulation of the CT modulated arousal in the two anaesthetized monkeys. Adapted from Figure 2 of [1], originally published under CC-BY license.

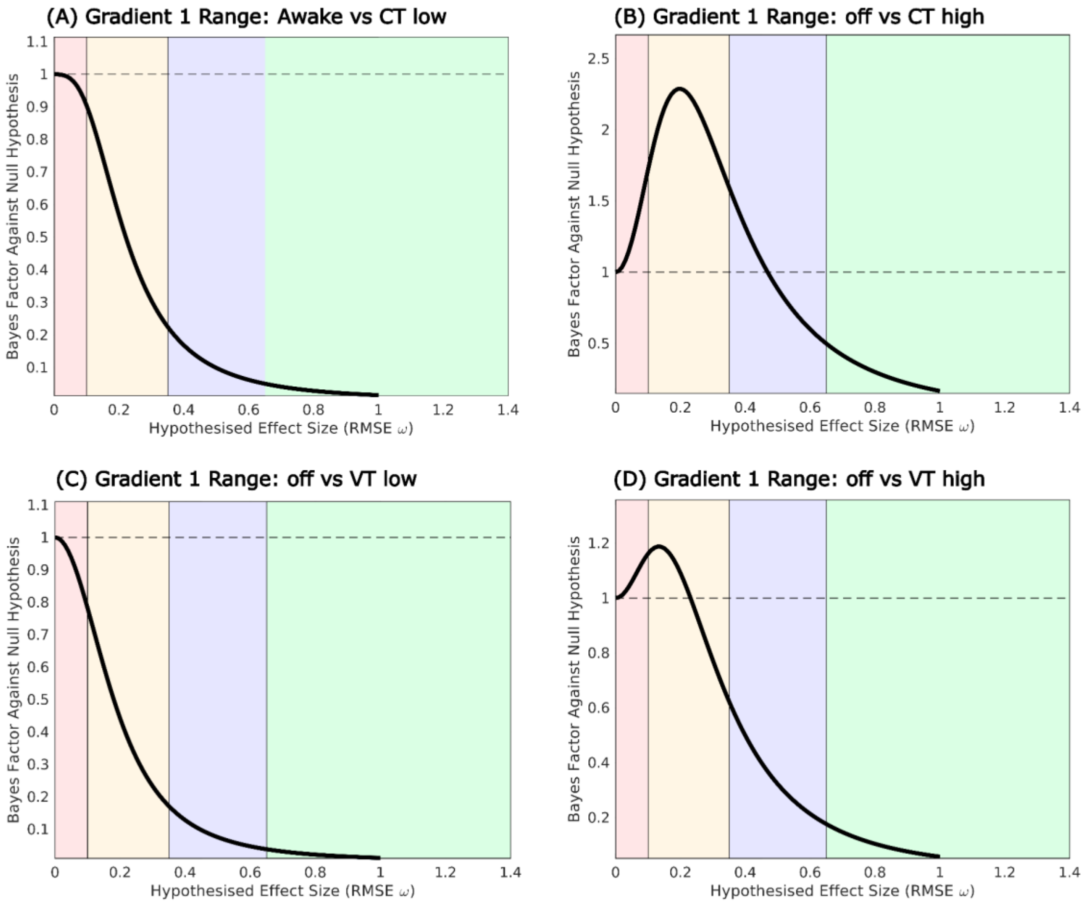

Figure S3. Bayes Factor Functions for the principal functional gradient of FC. (A) Awake versus low-intensity CT stimulation. (B) No stimulation anaesthesia (“off”) versus high-intensity CT stimulation. (C) No stimulation anaesthesia (“off”) versus low-intensity VT stimulation. (D) No stimulation anaesthesia (“off”) versus high-intensity VT stimulation. Bayes factors are displayed as odds in favour of the alternative hypothesis. The vertical axis is displayed on the logarithmic scale. The horizontal line denotes a Bayes factor of 1.0 (equal odds in favour of the alternative and null hypotheses). The red, orange, blue, and green zones in this figure are arbitrarily coloured following the same convention as in [2] and correspond to very small (0, 0.1), small (0.1, 0.35), medium (0.35, 0.65), and large (> 0.65) standardized effect sizes, respectively.

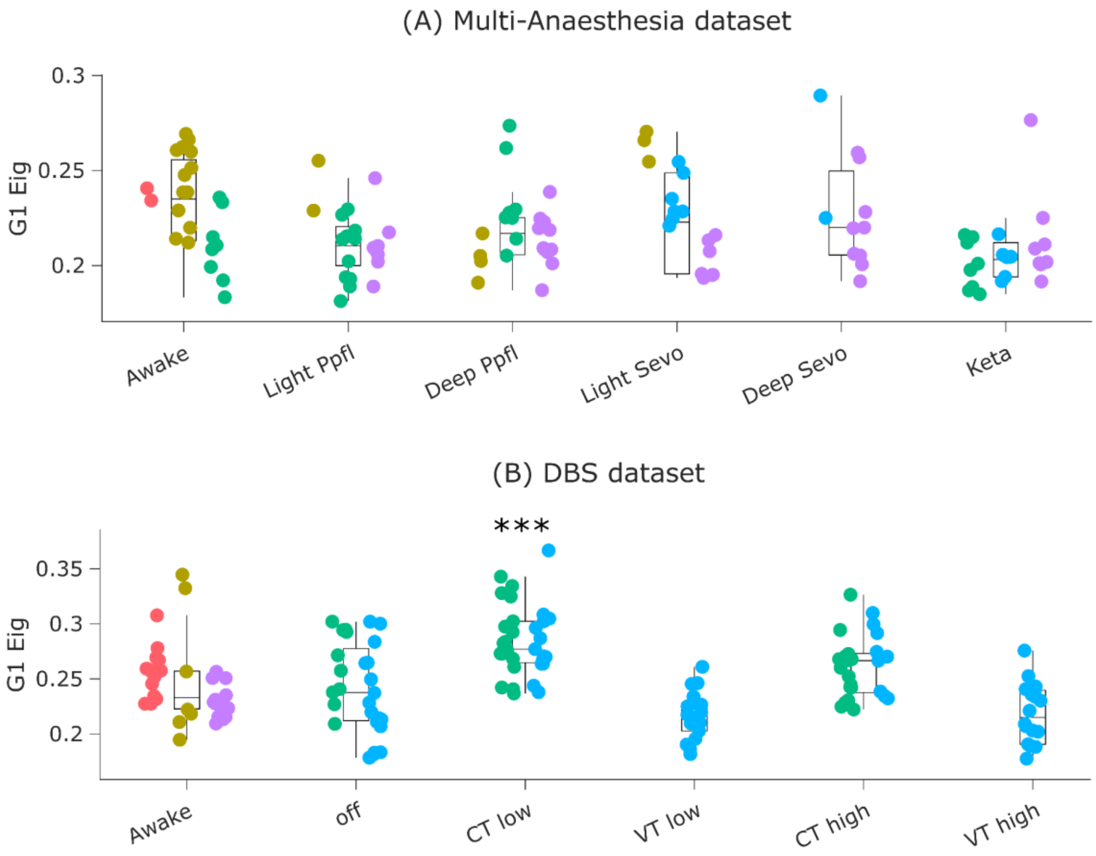

Figure S4. Influence of different anaesthetics (A) and DBS (B) on the relative importance of the principal gradient of functional connectivity (G1 Eig, quantified as ratio of the corresponding eigenvalue over the sum of all eigenvalues). ***, $p < 0.001$ (two-sided, FDR-corrected) against the no-stimulation ("off") anaesthesia condition, for the DBS dataset. Box plots: central line, median; box limits, upper and lower quartiles; whiskers, 1.5× interquartile range; within each panel, dots of the same colour are provided by the same animal. Multi-anaesthesia dataset: N=24 runs from 3 animals for Awake; 18 runs from 3 animals for Light Sevoflurane; 21 runs from 3 animals for Light Propofol; 11 runs from 2 animals for Deep Sevoflurane; 23 runs from 3 animals for Deep Propofol; 22 runs from 3 animals for Ketamine anaesthesia. DBS dataset: N=36 runs from 3 animals for Awake; 28 runs from 2 animals for anaesthesia (DBS-off); 31 runs from 2 animals for low amplitude centro-median thalamic DBS; 25 runs from 2 animals for high amplitude centro-median thalamic DBS; 18 runs from 1 animal for low amplitude ventro-lateral thalamic DBS; 18 runs from 1 animal for high amplitude ventro-lateral thalamic DBS. Source data are provided as a Source Data file.

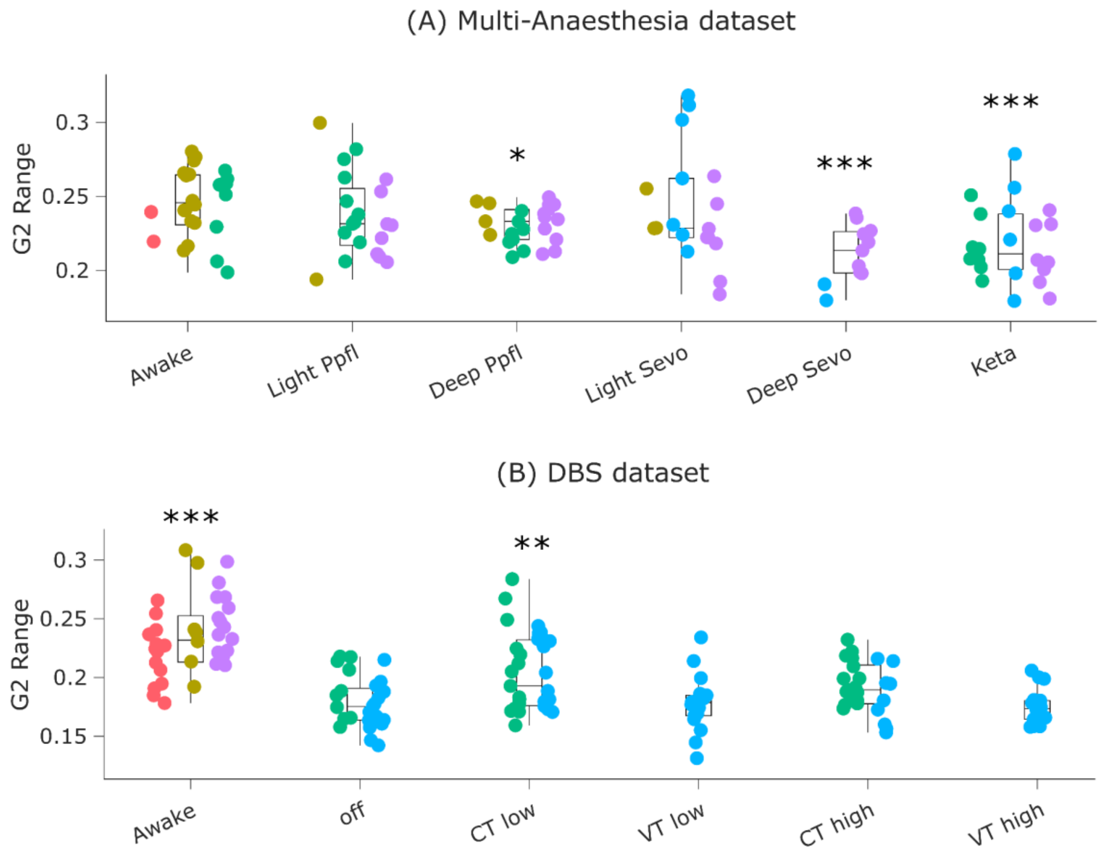

Figure S5. Influence of different anaesthetics (A) and DBS (B) on the second gradient of functional connectivity. *, $p < 0.05$; **, $p < 0.01$; ***, $p < 0.001$ (from linear mixed-effects modelling; two-sided, FDR-corrected) against the Awake condition in the Multi-Anaesthesia dataset (A) and against the no-stimulation ("off") anaesthesia condition in the DBS dataset (B). Box plots indicate the median and interquartile range of the distribution; within each panel, dots of the same colour are provided by the same animal. Multi-anaesthesia dataset: N=24 runs from 3 animals for Awake; 18 runs from 3 animals for Light Sevoflurane; 21 runs from 3 animals for Light Propofol; 11 runs from 2 animals for Deep Sevoflurane; 23 runs from 3 animals for Deep Propofol; 22 runs from 3 animals for Ketamine anaesthesia. DBS dataset: N=36 runs from 3 animals for Awake; 28 runs from 2 animals for anaesthesia (DBS-off); 31 runs from 2 animals for low amplitude centro-median thalamic DBS; 25 runs from 2 animals for high amplitude centro-median thalamic DBS; 18 runs from 1 animal for low amplitude ventro-lateral thalamic DBS; 18 runs from 1 animal for high amplitude ventro-lateral thalamic DBS. Source data are provided as a Source Data file.

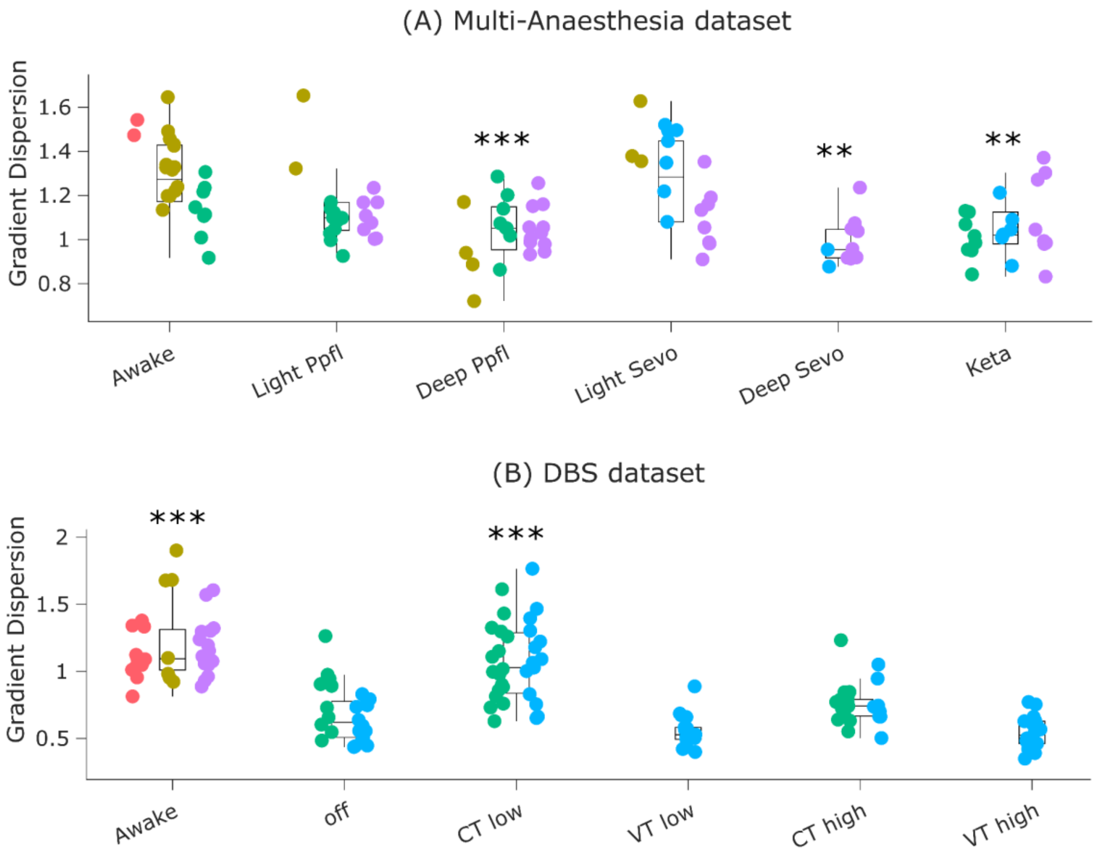

Figure S6. Influence of different anaesthetics (A) and DBS (B) on the dispersion of the first three gradients of functional connectivity. **, $p < 0.01$; ***, $p < 0.001$ (from linear mixed-effects modelling; two-sided, FDR-corrected) against the Awake condition in the Multi-Anaesthesia dataset (A) and against the no-stimulation ("off") anaesthesia condition in the DBS dataset (B). Box plots: central line, median; box limits, upper and lower quartiles; whiskers, 1.5× interquartile range; within each panel, dots of the same colour are provided by the same animal. Multi-anaesthesia dataset: N=24 runs from 3 animals for Awake; 18 runs from 3 animals for Light Sevoflurane; 21 runs from 3 animals for Light Propofol; 11 runs from 2 animals for Deep Sevoflurane; 23 runs from 3 animals for Deep Propofol; 22 runs from 3 animals for Ketamine anaesthesia. DBS dataset: N=36 runs from 3 animals for Awake; 28 runs from 2 animals for anaesthesia (DBS-off); 31 runs from 2 animals for low amplitude centro-median thalamic DBS; 25 runs from 2 animals for high amplitude centro-median thalamic DBS; 18 runs from 1 animal for low amplitude ventro-lateral thalamic DBS; 18 runs from 1 animal for high amplitude ventro-lateral thalamic DBS. Source data are provided as a Source Data file.

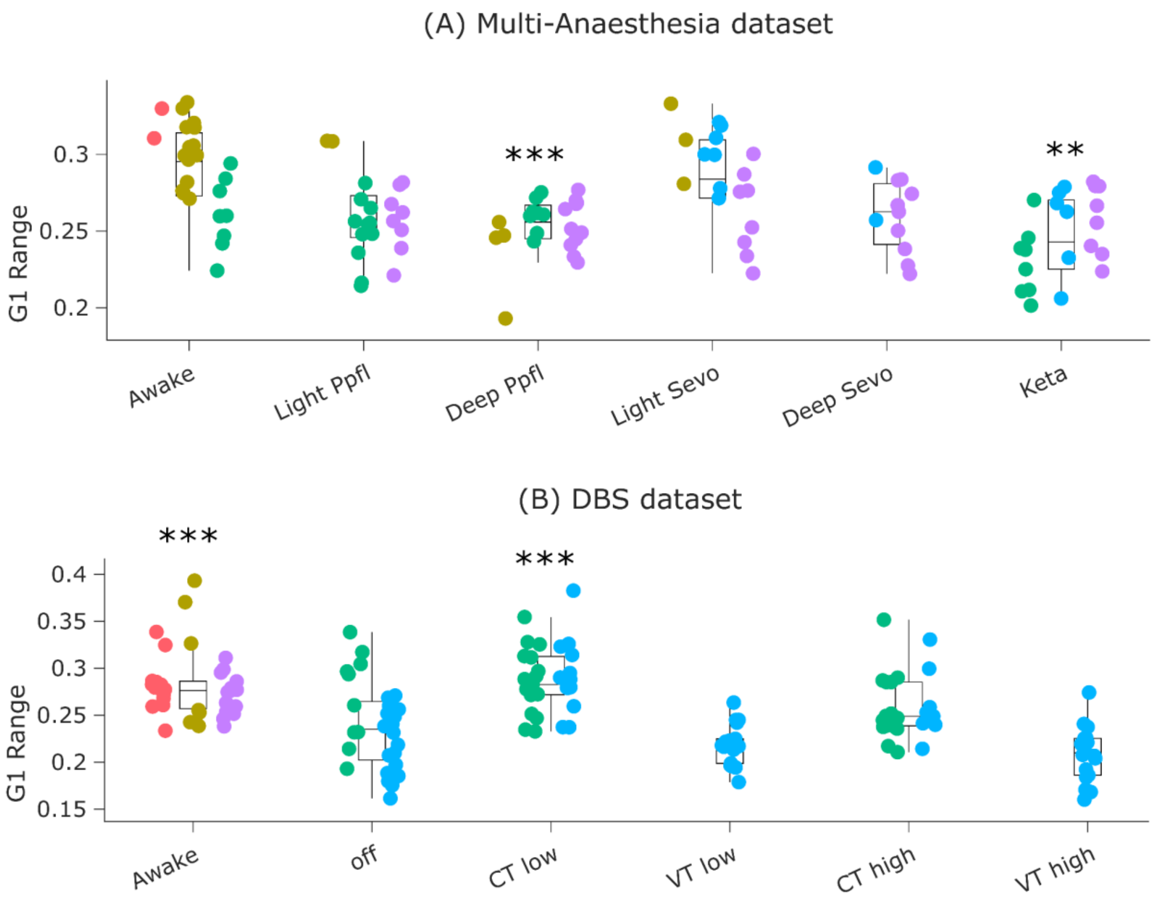

Figure S7. Influence of different anaesthetics (A) and DBS (B) on the range of the principal gradient of functional connectivity, setting the diffusion parameter alpha to 0.1. ** $p < 0.01$; ***, $p < 0.001$ (from linear mixed-effects modelling; two-sided, FDR-corrected) against the Awake condition in the Multi-Anaesthesia dataset (A) and against the no-stimulation ("off") anaesthesia condition in the DBS dataset (B). Box plots: central line, median; box limits, upper and lower quartiles; whiskers, 1.5× interquartile range; within each panel, dots of the same colour are provided by the same animal. Multi-anaesthesia dataset: N=24 runs from 3 animals for Awake; 18 runs from 3 animals for Light Sevoflurane; 21 runs from 3 animals for Light Propofol; 11 runs from 2 animals for Deep Sevoflurane; 23 runs from 3 animals for Deep Propofol; 22 runs from 3 animals for Ketamine anaesthesia. DBS dataset: N=36 runs from 3 animals for Awake; 28 runs from 2 animals for anaesthesia (DBS-off); 31 runs from 2 animals for low amplitude centro-median thalamic DBS; 25 runs from 2 animals for high amplitude centro-median thalamic DBS; 18 runs from 1 animal for low amplitude ventro-lateral thalamic DBS; 18 runs from 1 animal for high amplitude ventro-lateral thalamic DBS. Source data are provided as a Source Data file.

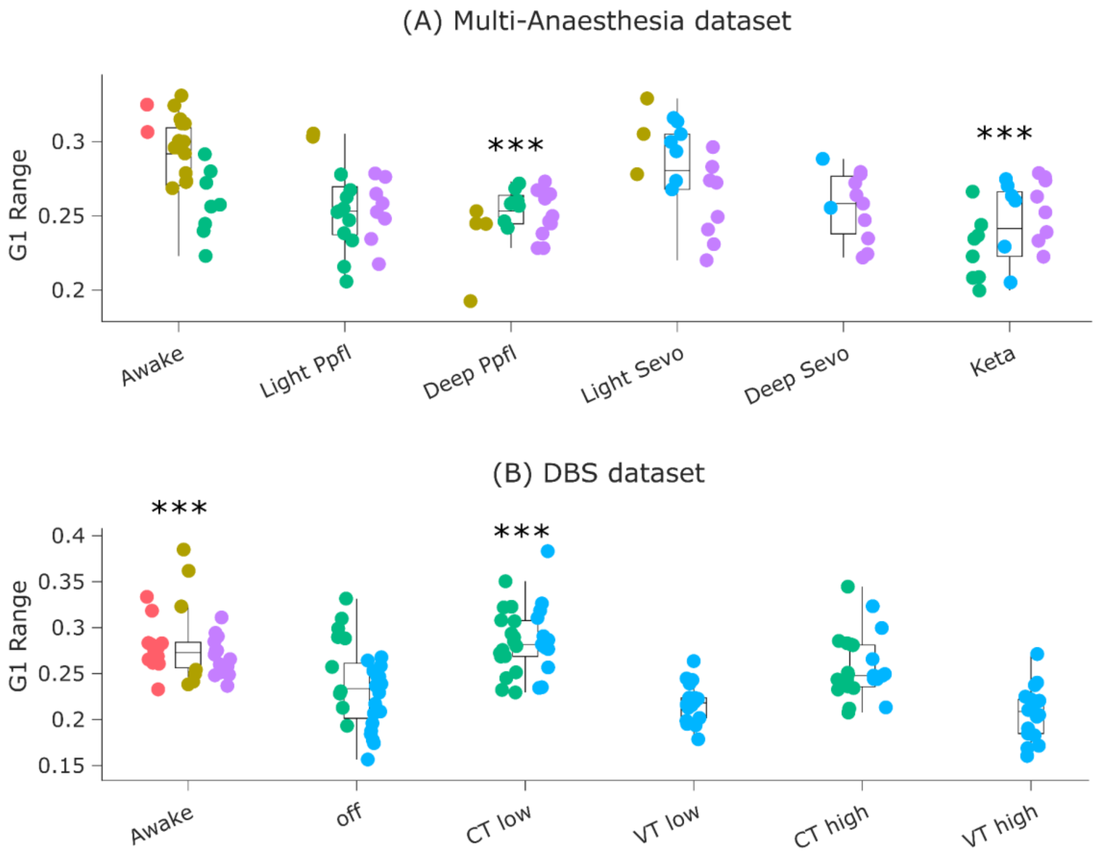

Figure S8. Influence of different anaesthetics (A) and DBS (B) on the range of the principal gradient of functional connectivity, setting the diffusion parameter alpha to 0.9. ***, $p < 0.001$ (from linear mixed-effects modelling; two-sided, FDR-corrected) against the Awake condition in the Multi-Anaesthesia dataset (A) and against the no-stimulation ("off") anaesthesia condition in the DBS dataset (B). Box plots: central line, median; box limits, upper and lower quartiles; whiskers, 1.5× interquartile range; within each panel, dots of the same colour are provided by the same animal. Multi-anaesthesia dataset: N=24 runs from 3 animals for Awake; 18 runs from 3 animals for Light Sevoflurane; 21 runs from 3 animals for Light Propofol; 11 runs from 2 animals for Deep Sevoflurane; 23 runs from 3 animals for Deep Propofol; 22 runs from 3 animals for Ketamine anaesthesia. DBS dataset: N=36 runs from 3 animals for Awake; 28 runs from 2 animals for anaesthesia (DBS-off); 31 runs from 2 animals for low amplitude centro-median thalamic DBS; 25 runs from 2 animals for high amplitude centro-median thalamic DBS; 18 runs from 1 animal for low amplitude ventro-lateral thalamic DBS; 18 runs from 1 animal for high amplitude ventro-lateral thalamic DBS. Source data are provided as a Source Data file.

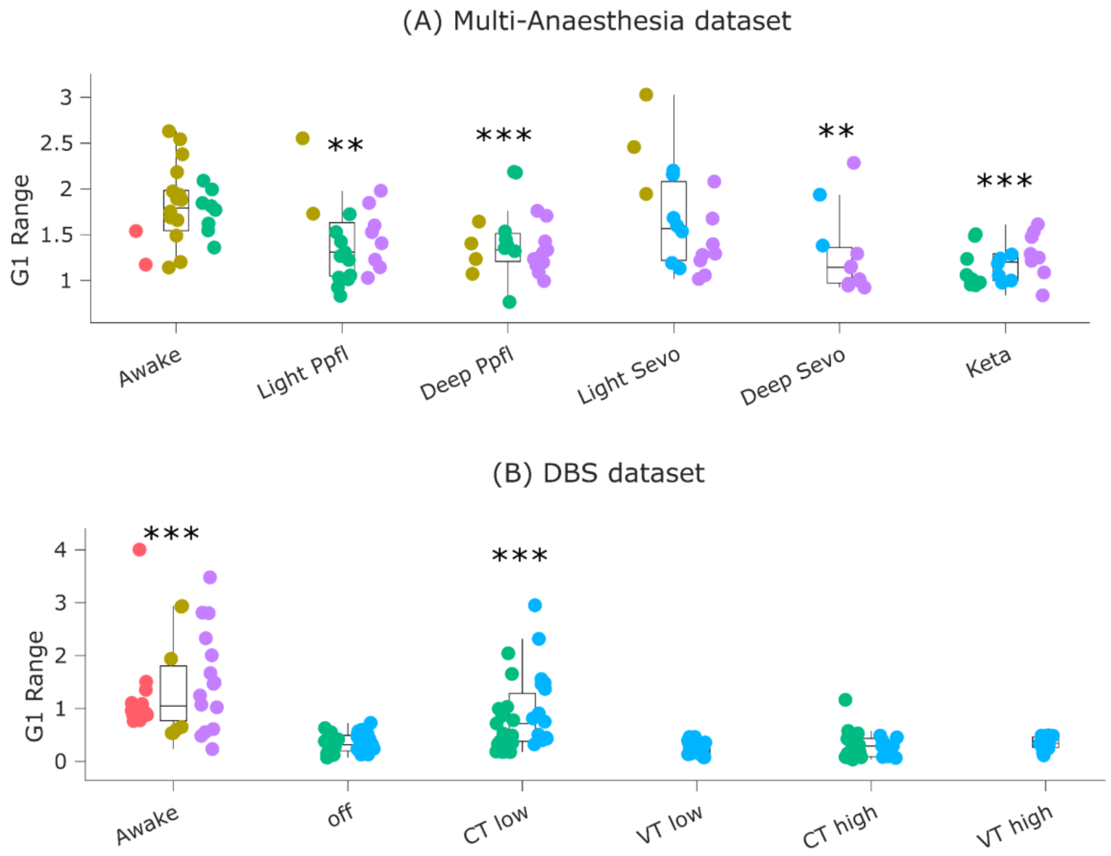

Figure S9. Influence of different anaesthetics (A) and DBS (B) on the range of the principal gradient of functional connectivity, using a density parameter of 50%. **, $p < 0.01$; ***, $p < 0.001$ (from linear mixed-effects modelling; two-sided, FDR-corrected) against the Awake condition in the Multi-Anaesthesia dataset (A) and against the no-stimulation ("off") anaesthesia condition in the DBS dataset (B). Box plots: central line, median; box limits, upper and lower quartiles; whiskers, 1.5× interquartile range; within each panel, dots of the same colour are provided by the same animal. Multi-anaesthesia dataset: N=24 runs from 3 animals for Awake; 18 runs from 3 animals for Light Sevoflurane; 21 runs from 3 animals for Light Propofol; 11 runs from 2 animals for Deep Sevoflurane; 23 runs from 3 animals for Deep Propofol; 22 runs from 3 animals for Ketamine anaesthesia. DBS dataset: N=36 runs from 3 animals for Awake; 28 runs from 2 animals for anaesthesia (DBS-off); 31 runs from 2 animals for low amplitude centro-median thalamic DBS; 25 runs from 2 animals for high amplitude centro-median thalamic DBS; 18 runs from 1 animal for low amplitude ventro-lateral thalamic DBS; 18 runs from 1 animal for high amplitude ventro-lateral thalamic DBS. Source data are provided as a Source Data file.

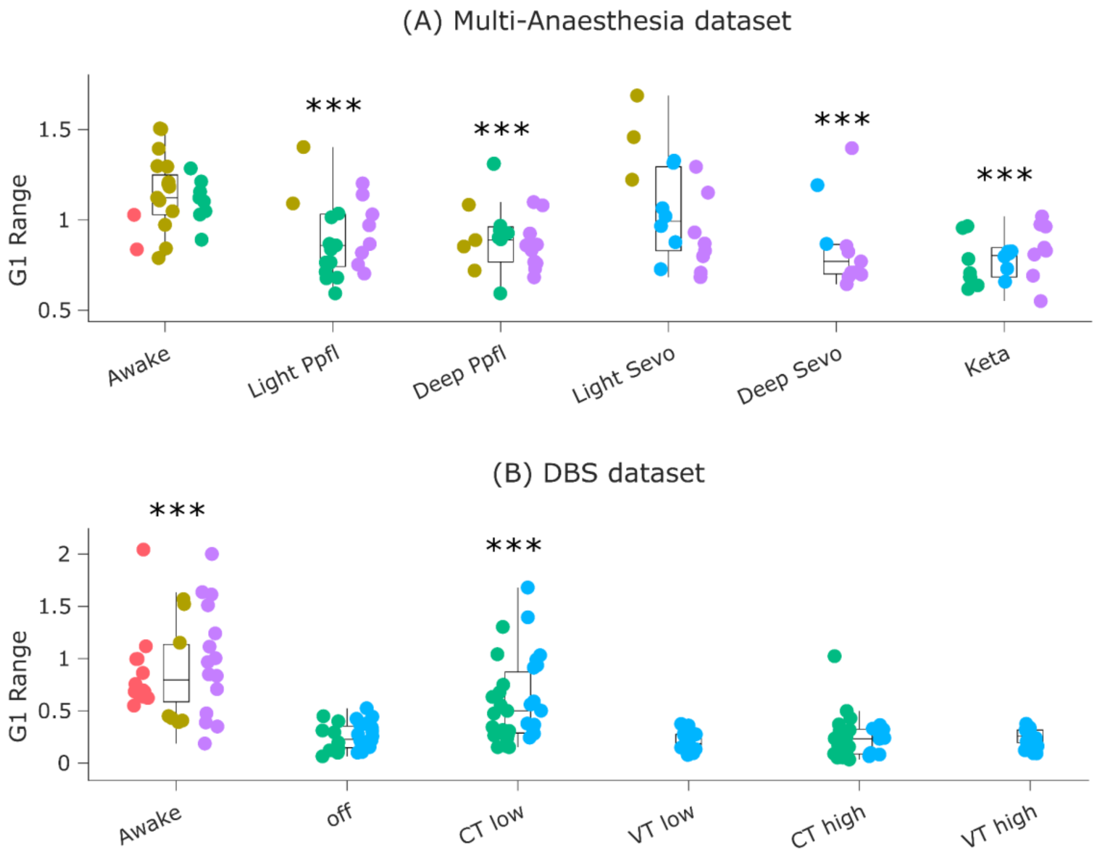

Figure S10. Influence of different anaesthetics (A) and DBS (B) on the range of the principal gradient of functional connectivity, using a density parameter of 90%. ***, p < 0.001 (from linear mixed-effects modelling; two-sided, FDR-corrected) against the Awake condition in the Multi-Anaesthesia dataset (A) and against the no-stimulation ("off") anaesthesia condition in the DBS dataset (B). Box plots: central line, median; box limits, upper and lower quartiles; whiskers, 1.5× interquartile range; within each panel, dots of the same colour are provided by the same animal. Multi-anaesthesia dataset: N=24 runs from 3 animals for Awake; 18 runs from 3 animals for Light Sevoflurane; 21 runs from 3 animals for Light Propofol; 11 runs from 2 animals for Deep Sevoflurane; 23 runs from 3 animals for Deep Propofol; 22 runs from 3 animals for Ketamine anaesthesia. DBS dataset: N=36 runs from 3 animals for Awake; 28 runs from 2 animals for anaesthesia (DBS-off); 31 runs from 2 animals for low amplitude centro-median thalamic DBS; 25 runs from 2 animals for high amplitude centro-median thalamic DBS; 18 runs from 1 animal for low amplitude ventro-lateral thalamic DBS; 18 runs from 1 animal for high amplitude ventro-lateral thalamic DBS. Source data are provided as a Source Data file.

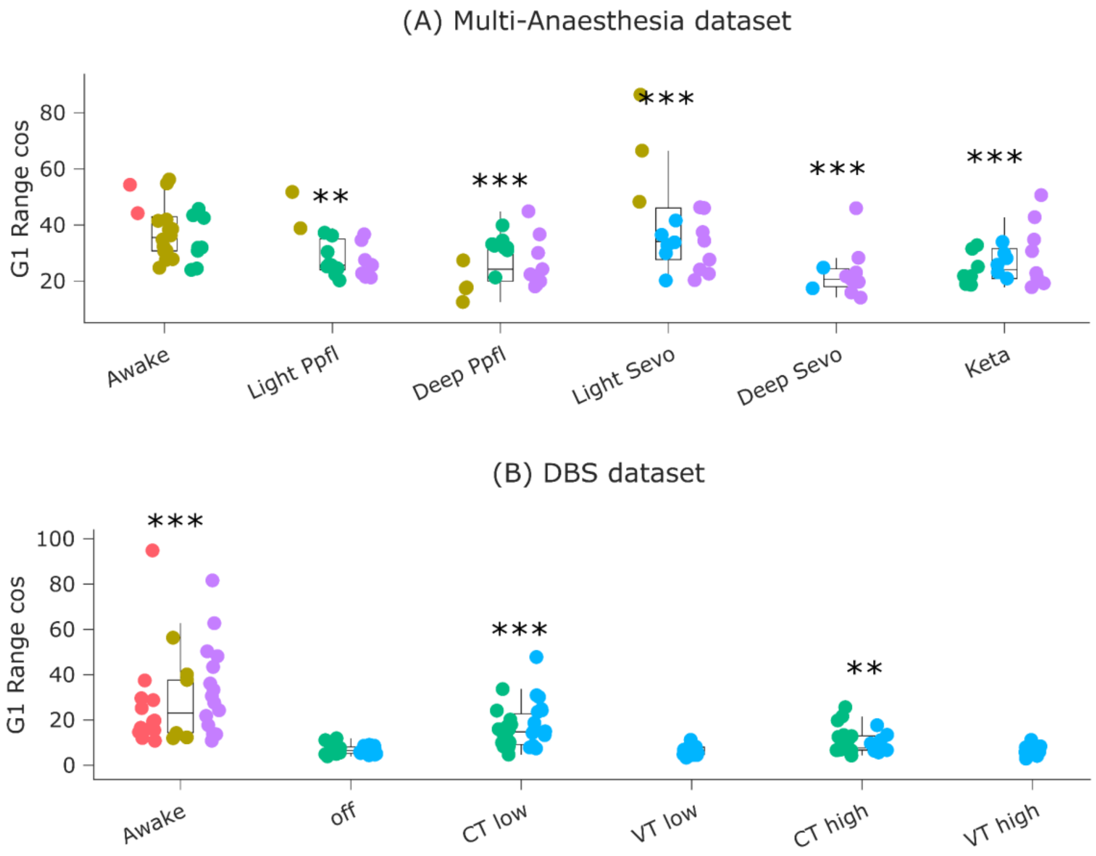

Figure S11. Influence of different anaesthetics (A) and DBS (B) on the range of the principal gradient of functional connectivity, using cosine similarity as the kernel. **, $p < 0.01$; ***, $p < 0.001$ (from linear mixed-effects modelling; two-sided, FDR-corrected) against the Awake condition in the Multi-Anaesthesia dataset (A) and against the no-stimulation ("off") anaesthesia condition in the DBS dataset (B). Box plots: central line, median; box limits, upper and lower quartiles; whiskers, 1.5× interquartile range; within each panel, dots of the same colour are provided by the same animal. Multi-anaesthesia dataset: N=24 runs from 3 animals for Awake; 18 runs from 3 animals for Light Sevoflurane; 21 runs from 3 animals for Light Propofol; 11 runs from 2 animals for Deep Sevoflurane; 23 runs from 3 animals for Deep Propofol; 22 runs from 3 animals for Ketamine anaesthesia. DBS dataset: N=36 runs from 3 animals for Awake; 28 runs from 2 animals for anaesthesia (DBS-off); 31 runs from 2 animals for low amplitude centro-median thalamic DBS; 25 runs from 2 animals for high amplitude centro-median thalamic DBS; 18 runs from 1 animal for low amplitude ventro-lateral thalamic DBS; 18 runs from 1 animal for high amplitude ventro-lateral thalamic DBS. Source data are provided as a Source Data file.

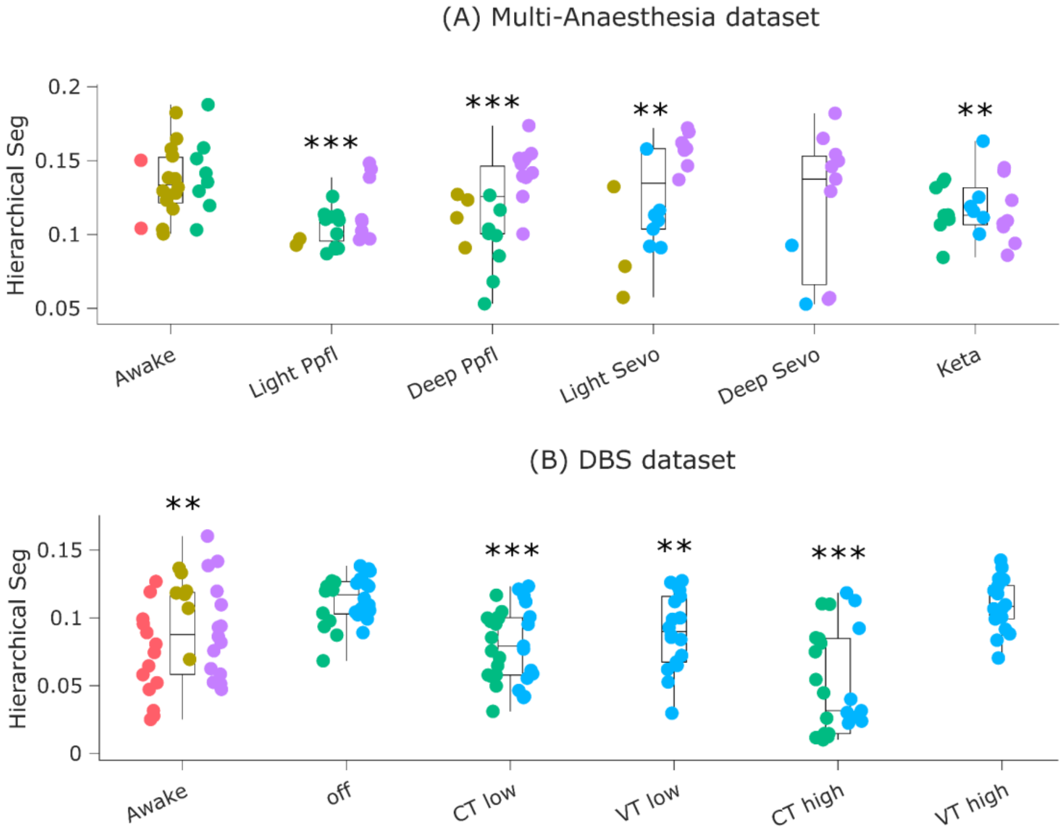

Figure S12. Influence of different anaesthetics (A) and DBS (B) on hierarchical segregation of the macaque brain. **, $p < 0.01$; ***, $p < 0.001$ (from linear mixed-effects modelling; two-sided, FDR-corrected) against the Awake condition in the Multi-Anaesthesia dataset (A) and against the no-stimulation ("off") anaesthesia condition in the DBS dataset (B). Box plots: central line, median; box limits, upper and lower quartiles; whiskers, 1.5× interquartile range; within each panel, dots of the same colour are provided by the same animal. Multi-anaesthesia dataset: N=24 runs from 3 animals for Awake; 18 runs from 3 animals for Light Sevoflurane; 21 runs from 3 animals for Light Propofol; 11 runs from 2 animals for Deep Sevoflurane; 23 runs from 3 animals for Deep Propofol; 22 runs from 3 animals for Ketamine anaesthesia. DBS dataset: N=36 runs from 3 animals for Awake; 28 runs from 2 animals for anaesthesia (DBS-off); 31 runs from 2 animals for low amplitude centro-median thalamic DBS; 25 runs from 2 animals for high amplitude centro-median thalamic DBS; 18 runs from 1 animal for low amplitude ventro-lateral thalamic DBS; 18 runs from 1 animal for high amplitude ventro-lateral thalamic DBS. Source data are provided as a Source Data file.

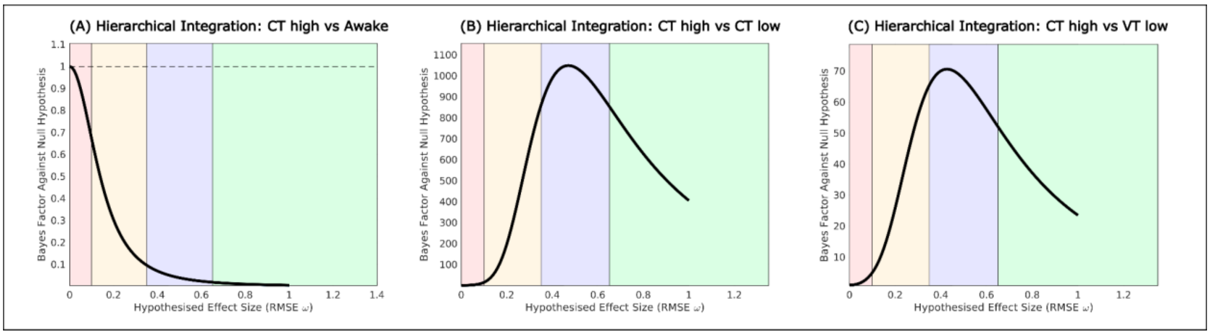

Figure S13. Bayes Factor Functions for Hierarchical Integration. (A) Awake versus high-intensity CT stimulation. (B) High-intensity CT stimulation versus low-intensity CT stimulation. (C) High-intensity CT stimulation versus low-intensity VT stimulation. Bayes factors are displayed as odds in favour of the alternative hypothesis. The vertical axis is displayed on the logarithmic scale. The horizontal line denotes a Bayes factor of 1.0 (equal odds in favour of the alternative and null hypotheses). The red, orange, blue, and green zones in this figure are arbitrarily coloured following the same convention as in [2] and correspond to very small (0, 0.1), small (0.1, 0.35), medium (0.35, 0.65), and large (> 0.65) standardized effect sizes, respectively.

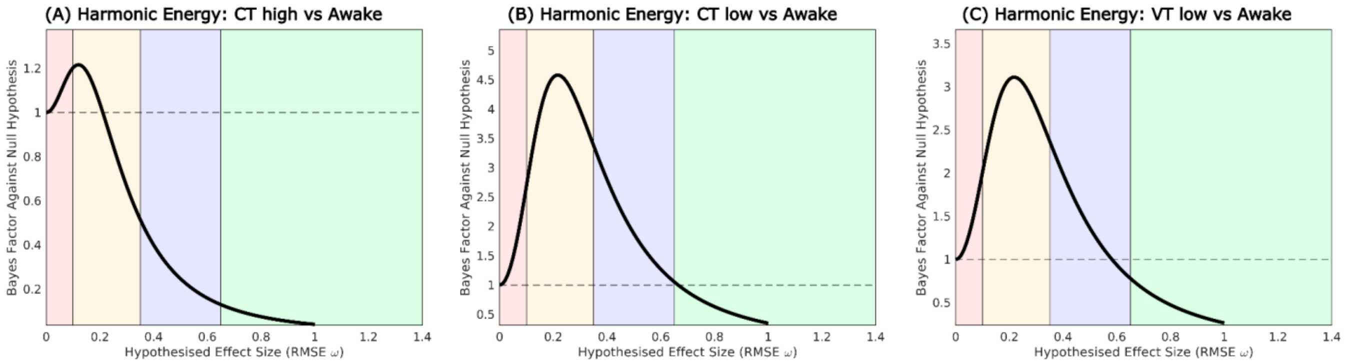

Figure S14. Bayes Factor Functions for Harmonic Energy. (A) Awake versus high-intensity CT stimulation. (B) Awake versus low-intensity CT stimulation. (C) Awake versus low-intensity VT stimulation. Bayes factors are displayed as odds in favour of the alternative hypothesis. The vertical axis is displayed on the logarithmic scale. The horizontal line denotes a Bayes factor of 1.0 (equal odds in favour of the alternative and null hypotheses). The red, orange, blue, and green zones in this figure are arbitrarily coloured following the same convention as in [2] and correspond to very small (0, 0.1), small (0.1, 0.35), medium (0.35, 0.65), and large (> 0.65) standardized effect sizes, respectively.

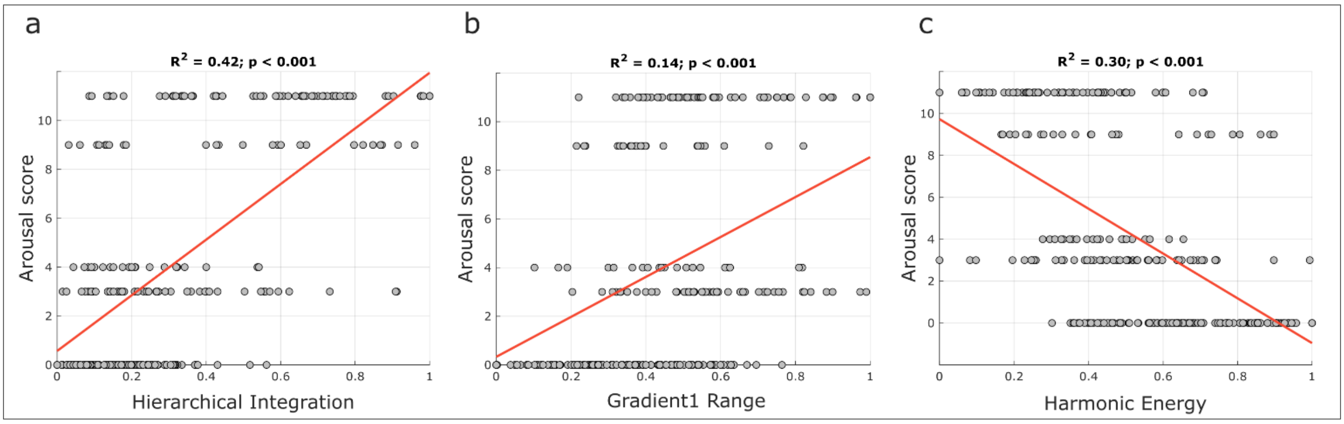

Figure S15. Regression lines between behavioural arousal score and neural predictors, from the combined Multi-Anaesthesia and DBS datasets. (a) Hierarchical integration. (b) Gradient 1 range. (c) Harmonic energy. Each data-point represents one run from one animal. Multi-anaesthesia dataset: N=24 runs from 3 animals for Awake; 18 runs from 3 animals for Light Sevoflurane; 21 runs from 3 animals for Light Propofol; 11 runs from 2 animals for Deep Sevoflurane; 23 runs from 3 animals for Deep Propofol; 22 runs from 3 animals for Ketamine anaesthesia. DBS dataset: N=36 runs from 3 animals for Awake; 28 runs from 2 animals for anaesthesia (DBS-off); 31 runs from 2 animals for low amplitude centro-median thalamic DBS; 25 runs from 2 animals for high amplitude centro-median thalamic DBS; 18 runs from 1 animal for low amplitude ventro-lateral thalamic DBS; 18 runs from 1 animal for high amplitude ventro-lateral thalamic DBS.

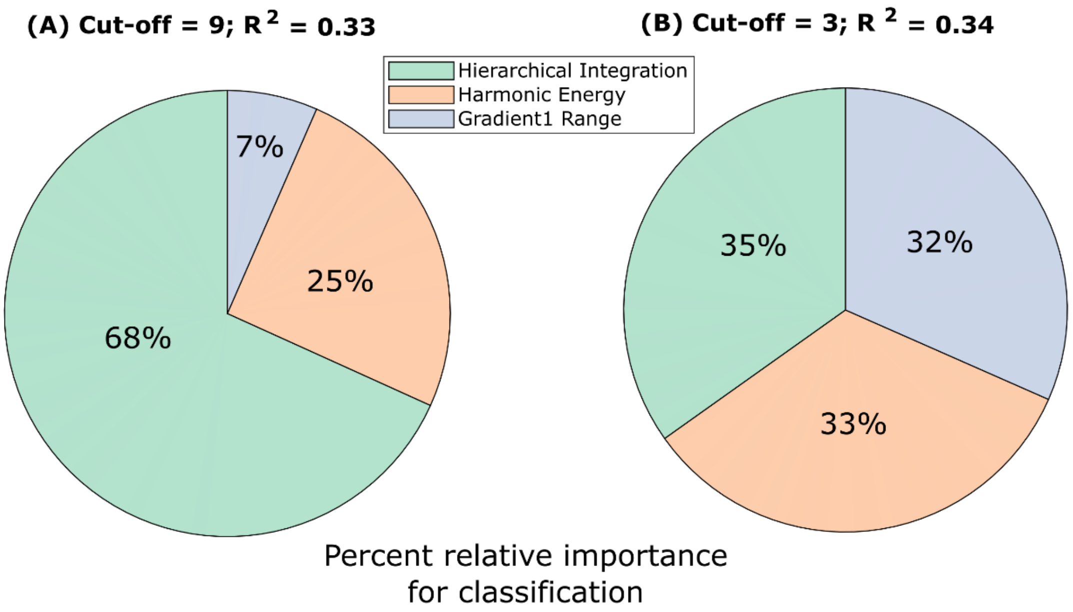

Figure S16. Dominance analysis for classification of arousal scores based on different cut-off values, from the combined Multi-Anaesthesia and DBS datasets. (A) Cut-off of 9 separates wakefulness and high-amplitude CT stimulation from all other conditions. (B) Cut-off of 3 separates wakefulness and both high- and low-amplitude CT stimulation, from anaesthesia with different drugs and VT stimulation. Pie charts represent the percentage of relative importance of each predictor from dominance analysis.

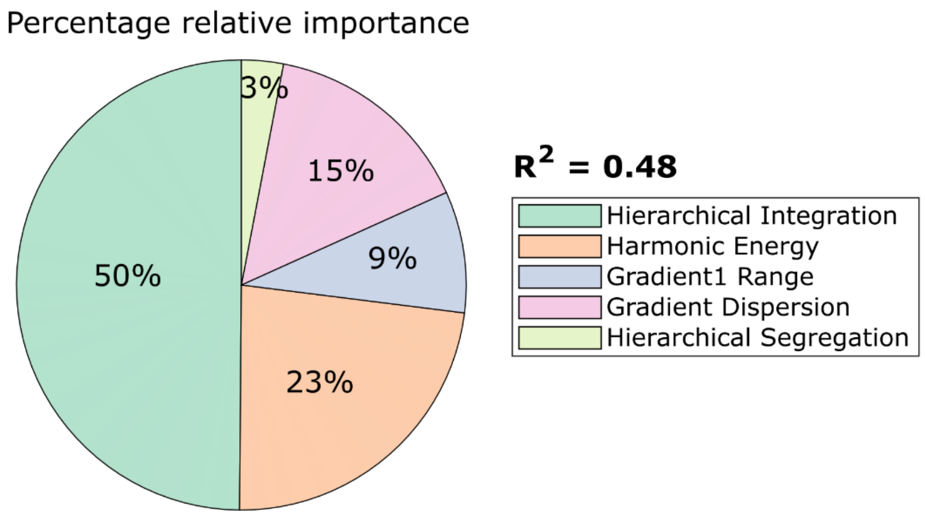

Figure S17. Dominance analysis with additional predictors, from the combined Multi-Anaesthesia and DBS datasets. The best two predictors remain the same, but gradient dispersion exhibits greater relative importance (15%) than the range of the first gradient alone (8%). Hierarchical segregation provides barely any contribution (3%).

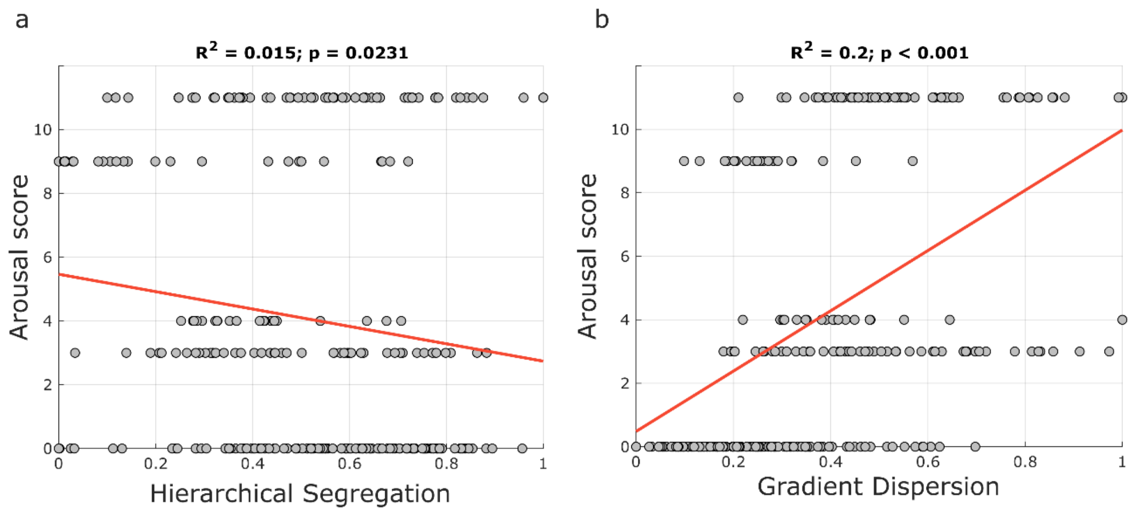

Figure S18. Regression lines between behavioural arousal score and additional neural predictors, from the combined Multi-Anaesthesia and DBS datasets. (a) Hierarchical segregation. (b) Gradient dispersion. Each data-point represents one run from one animal. Multi-anaesthesia dataset: N=24 runs from 3 animals for Awake; 18 runs from 3 animals for Light Sevoflurane; 21 runs from 3 animals for Light Propofol; 11 runs from 2 animals for Deep Sevoflurane; 23 runs from 3 animals for Deep Propofol; 22 runs from 3 animals for Ketamine anaesthesia. DBS dataset: N=36 runs from 3 animals for Awake; 28 runs from 2 animals for anaesthesia (DBS-off); 31 runs from 2 animals for low amplitude centro-median thalamic DBS; 25 runs from 2 animals for high amplitude centro-median thalamic DBS; 18 runs from 1 animal for low amplitude ventro-lateral thalamic DBS; 18 runs from 1 animal for high amplitude ventro-lateral thalamic DBS.

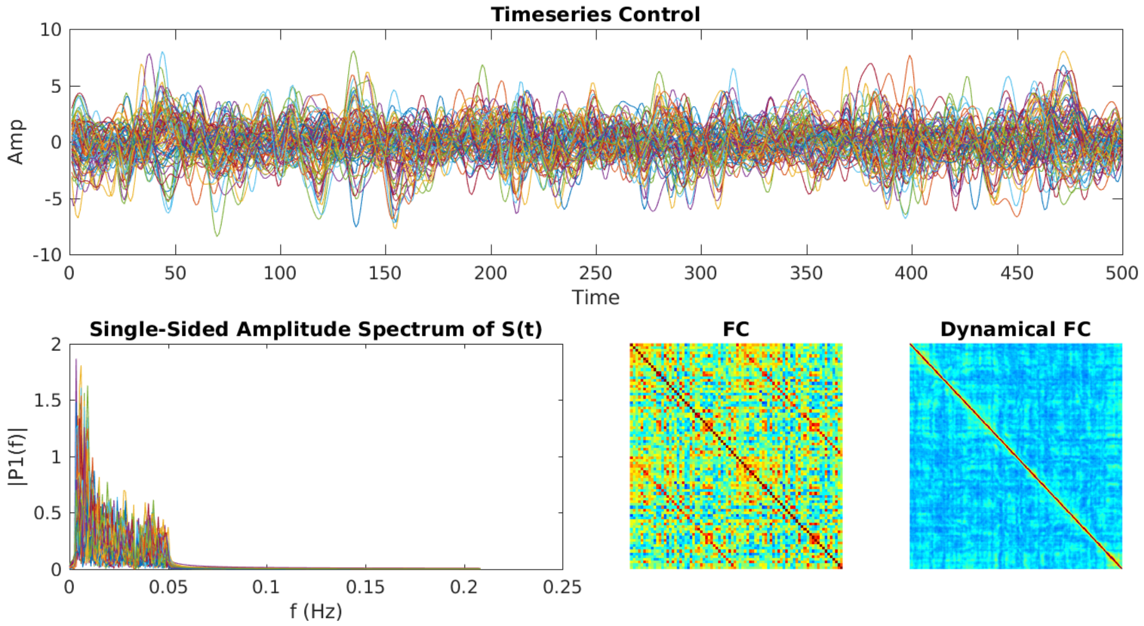

Figure S19. Example QC plot for an artefact-free trial. The preprocessed and denoised fMRI time-series are plotted, as well as the Fourier spectrum (showing frequencies in the entire range admitted by the band-pass filter), functional connectivity (showing the expected higher correlation between homotopic regions in the two hemispheres, appearing as the two minor diagonals), and functional connectivity dynamics (showing the expected high correlation between consecutive time-points, appearing as a high-value diagonal).

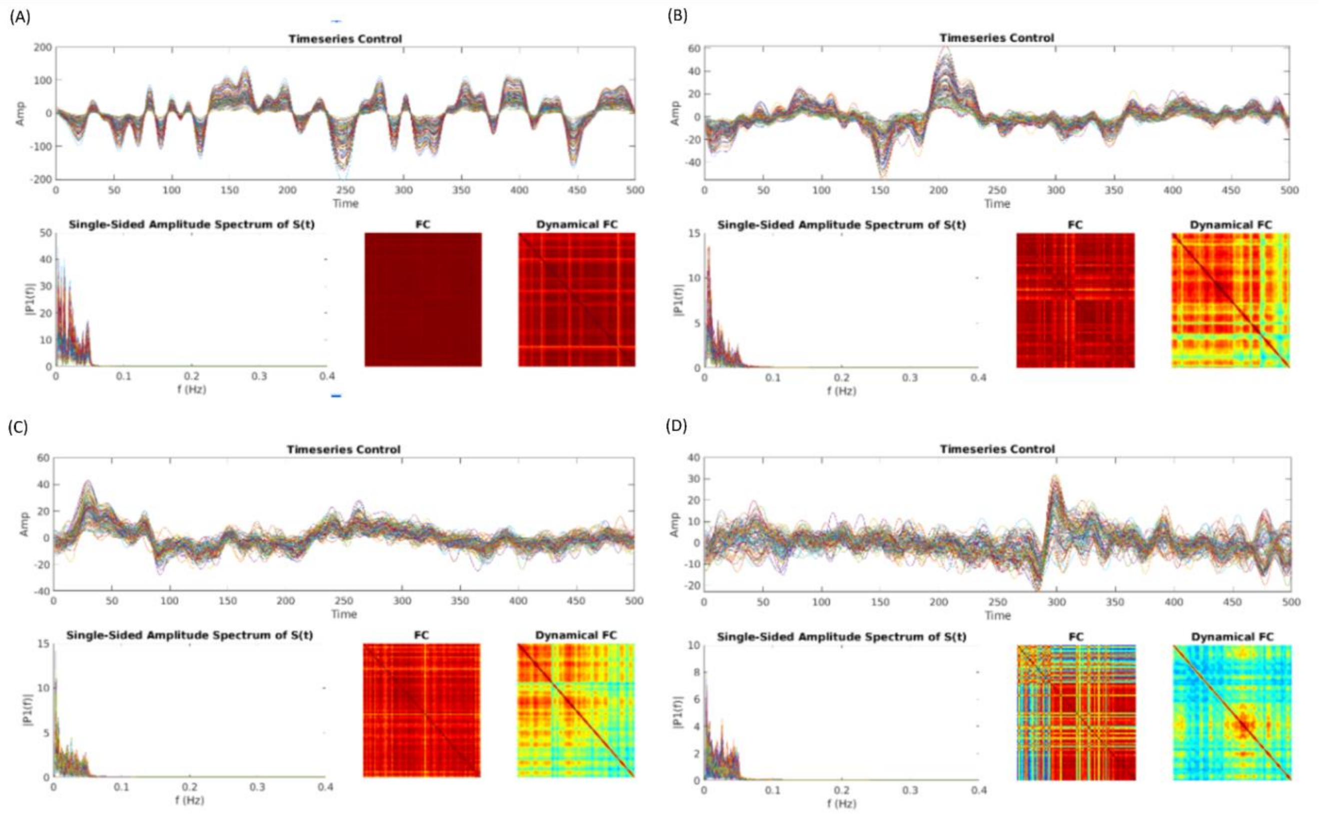

Figure S20. Example QC plot for rejected trials. (A) DBS dataset, Off condition, Run 1: Abnormal oscillatory patterns of activity. Extremely high and uniform FC and dFC values. Concentration is a few frequencies on the spectrum. (B) DBS dataset, Off condition, Run 5: Abnormal oscillatory patterns of activity. High FC and dFC connectivity values. Concentration is a few frequencies on the spectrum. (C) DBS dataset, Off condition, Run 34: Abnormal oscillatory patterns of activity. Peaks of activity around 30 timepoints. Abnormally high FC and dFC values. Concentration in a few frequencies on the spectrum. (D) DBS dataset, 5V CT stimulation condition, Run 15: Sinusoidal patterns of activity resulting in abnormally high FC. Artefactual peak of activity around 300 timepoints also visible as a zone of high dFC.

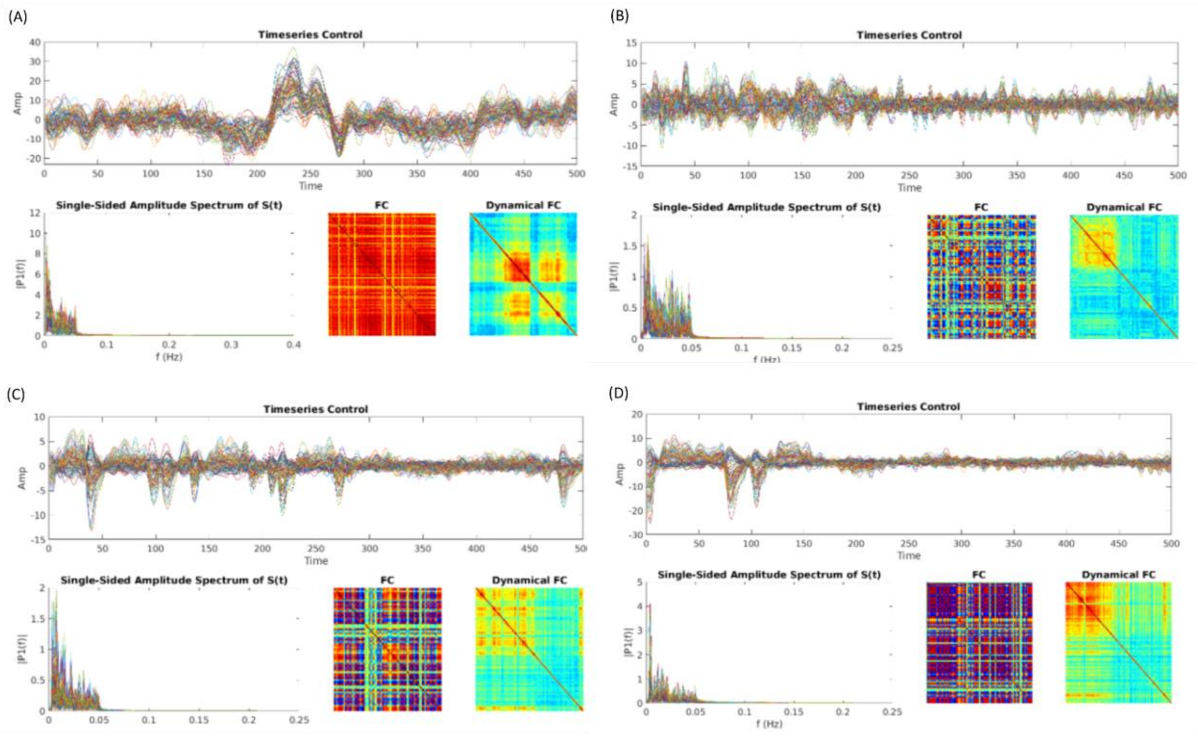

Figure S21. Example QC plot for rejected trials (continued). (A) DBS dataset, 5V CT stimulation condition, Run 27: Abnormal oscillatory patterns of activity (too much sinusoidal activity), biasing the FC to exhibit unusually high values, followed by a peak of activity around 200-300 timepoints, reflected in a zone of high dFC. (B) Multi-Anaesthesia dataset, Deep Propofol condition, Run 15: Abnormal burst of activity and sinusoidal waves in the first half of the recording, also clearly visible in the dFC. (C) Multi-Anaesthesia dataset, Light Sevoflurane condition, Run 7: Burst of activity and peaks visible in the dFC, and unusual correlation patterns in the FC, with extreme values. (D) Multi-Anaesthesia dataset, Deep Sevo condition, Run 19: Burst of activity with extreme peaks, leading to artifactually high dFC in the first half of the recording, and extreme FC values.

Supplementary References

1. Tasserie, J. *et al.* Deep brain stimulation of the thalamus restores signatures of consciousness in a nonhuman primate model. *Sci. Adv* **8**, 5547 (2022).

2. Johnson, V. E., Pramanik, S. & Shudde, R. Bayes factor functions for reporting outcomes of hypothesis tests. *Proceedings of the National Academy of Sciences* **120**, e2217331120 (2023).
