# Supplementary material for: Local orchestration of distributed functional patterns supporting loss and restoration of consciousness in the primate brain
Source: Nat Commun. 2024 Mar 11;15:2171. doi: 10.1038/s41467-024-46382-w (PMC10925605; doi:10.1038/s41467-024-46382-w)
Supplement: Supplementary file 3 — Description of Additional Supplementary Files [file 41467_2024_46382_MOESM3_ESM.pdf]

Description of Additional Supplementary Files

Supplementary Data 1.
Statistical results from linear mixed effects modelling and Bayes Factor Functions for the MultiAnaesthesia dataset, for the Gradient1 Range measure. BF10, Bayes Factor for the alternative over the null hypothesis.

Supplementary Data 2.
Statistical results from linear mixed effects modelling and Bayes Factor Functions for the DBS dataset, for the Gradient1 Range measure. BF10, Bayes Factor for the alternative over the null hypothesis.

Supplementary Data 3.
Statistical results from linear mixed effects modelling and Bayes Factor Functions for the MultiAnaesthesia dataset, for the Gradient1 Eigenvalue importance measure. BF10, Bayes Factor for the alternative over the null hypothesis.

Supplementary Data 4.
Statistical results from linear mixed effects modelling and Bayes Factor Functions for the DBS dataset, for the Gradient1 Eigenvalue importance measure. BF10, Bayes Factor for the alternative over the null hypothesis.

Supplementary Data 5.
Statistical results from linear mixed effects modelling and Bayes Factor Functions for the MultiAnaesthesia dataset, for the Gradient2 Range measure. BF10, Bayes Factor for the alternative over the null hypothesis.

Supplementary Data 6.
Statistical results from linear mixed effects modelling and Bayes Factor Functions for the DBS dataset, for the Gradient2 Range measure. BF10, Bayes Factor for the alternative over the null hypothesis.

Supplementary Data 7.
Statistical results from linear mixed effects modelling and Bayes Factor Functions for the MultiAnaesthesia dataset, for the Gradient Dispersion measure. BF10, Bayes Factor for the alternative over the null hypothesis.

Supplementary Data 8.
Statistical results from linear mixed effects modelling and Bayes Factor Functions for the DBS dataset, for the Gradient Dispersion measure. BF10, Bayes Factor for the alternative over the null hypothesis.

Supplementary Data 9.
Statistical results from linear mixed effects modelling and Bayes Factor Functions for the MultiAnaesthesia dataset, for the Gradient1 Range measure with alpha parameter = 0.1. BF10, Bayes Factor for the alternative over the null hypothesis.

Supplementary Data 10.
Statistical results from linear mixed effects modelling and Bayes Factor Functions for the DBS dataset, for the Gradient1 Range measure with alpha parameter = 0.1. BF10, Bayes Factor for the alternative over the null hypothesis.

Supplementary Data 11.
Statistical results from linear mixed effects modelling and Bayes Factor Functions for the MultiAnaesthesia dataset, for the Gradient1 Range measure with alpha parameter = 0.9. BF10, Bayes Factor for the alternative over the null hypothesis.

Supplementary Data 12.
Statistical results from linear mixed effects modelling and Bayes Factor Functions for the DBS dataset, for the Gradient1 Range measure with alpha parameter = 0.9. BF10, Bayes Factor for the alternative over the null hypothesis.

Supplementary Data 13.
Statistical results from linear mixed effects modelling and Bayes Factor Functions for the MultiAnaesthesia dataset, for the Gradient1 Range measure with density parameter = 0.5. BF10, Bayes Factor for the alternative over the null hypothesis.

Supplementary Data 14.
Statistical results from linear mixed effects modelling and Bayes Factor Functions for the DBS dataset, for the Gradient1 Range measure with density parameter = 0.5. BF10, Bayes Factor for the alternative over the null hypothesis.

Supplementary Data 15.
Statistical results from linear mixed effects modelling and Bayes Factor Functions for the MultiAnaesthesia dataset, for the Gradient1 Range measure with density parameter = 0.9. BF10, Bayes Factor for the alternative over the null hypothesis.

Supplementary Data 16.
Statistical results from linear mixed effects modelling and Bayes Factor Functions for the DBS dataset, for the Gradient1 Range measure with density parameter = 0.9. BF10, Bayes Factor for the alternative over the null hypothesis.

Supplementary Data 17.
Statistical results from linear mixed effects modelling and Bayes Factor Functions for the MultiAnaesthesia dataset, for the Gradient1 Range measure with cosine similarity kernel. BF10, Bayes Factor for the alternative over the null hypothesis.

Supplementary Data 18.
Statistical results from linear mixed effects modelling and Bayes Factor Functions for the DBS dataset, for the Gradient1 Range measure with cosine similarity kernel. BF10, Bayes Factor for the alternative over the null hypothesis.

Supplementary Data 19.
Statistical results from linear mixed effects modelling and Bayes Factor Functions for the MultiAnaesthesia dataset, for the Hierarchical Segregation measure. BF10, Bayes Factor for the alternative over the null hypothesis.

Supplementary Data 20.
Statistical results from linear mixed effects modelling and Bayes Factor Functions for the DBS dataset, for the Hierarchical Segregation measure. BF10, Bayes Factor for the alternative over the null hypothesis.

Supplementary Data 21.
Statistical results from linear mixed effects modelling and Bayes Factor Functions for the MultiAnaesthesia dataset, for the Hierarchical Integration measure. BF10, Bayes Factor for t he alternative over the null hypothesis.

Supplementary Data 22.
Statistical results from linear mixed effects modelling and Bayes Factor Functions for the DBS dataset, for the Hierarchical Integration measure. BF10, Bayes Factor for the alternative over the null hypothesis.

Supplementary Data 23.
Statistical results from linear mixed effects modelling and Bayes Factor Functions for the MultiAnaesthesia dataset, for the Harmonic Energy measure. BF10, Bayes Factor for the alternative over the null hypothesis.

Supplementary Data 24.
Statistical results from linear mixed effects modelling and Bayes Factor Functions for the DBS dataset, for the Harmonic Energy measure. BF10, Bayes Factor for the alternative over the null hypothesis.

Supplementary Data 25.
Parameters for anaesthesia and behavioural scores, for each animal and each anaesthetic used in the MultiAnaesthesia dataset. See Methods for the full description of the non-human primate behavioural arousal scale.
