# Supplementary material for: Local orchestration of distributed functional patterns supporting loss and restoration of consciousness in the primate brain
Source: Nat Commun. 2024 Mar 11;15:2171. doi: 10.1038/s41467-024-46382-w (PMC10925605; doi:10.1038/s41467-024-46382-w)
Supplement: Supplementary file 5 — Reporting Summary [file 41467_2024_46382_MOESM5_ESM.pdf]

nature portfolio

Corresponding author(s): Andrea Luppi; Rodrigo Cofre

Last updated by author(s): Jan 20, 2024

Reporting Summary

Nature Portfolio wishes to improve the reproducibility of the work that we publish. This form provides structure for consistency and transparency in reporting. For further information on Nature Portfolio policies, see our Editorial Policies and the Editorial Policy Checklist.

Statistics

For all statistical analyses, confirm that the following items are present in the figure legend, table legend, main text, or Methods section.

n/a	Confirmed	
☐	☒	The exact sample size (*n*) for each experimental group/condition, given as a discrete number and unit of measurement
☐	☒	A statement on whether measurements were taken from distinct samples or whether the same sample was measured repeatedly
☐	☒	The statistical test(s) used AND whether they are one- or two-sided<br>*Only common tests should be described solely by name; describe more complex techniques in the Methods section.*
☒	☐	A description of all covariates tested
☐	☒	A description of any assumptions or corrections, such as tests of normality and adjustment for multiple comparisons
☐	☒	A full description of the statistical parameters including central tendency (e.g. means) or other basic estimates (e.g. regression coefficient) AND variation (e.g. standard deviation) or associated estimates of uncertainty (e.g. confidence intervals)
☐	☒	For null hypothesis testing, the test statistic (e.g. *F*, *t*, *r*) with confidence intervals, effect sizes, degrees of freedom and *P* value noted<br>*Give P values as exact values whenever suitable.*
☐	☒	For Bayesian analysis, information on the choice of priors and Markov chain Monte Carlo settings
☒	☐	For hierarchical and complex designs, identification of the appropriate level for tests and full reporting of outcomes
☐	☒	Estimates of effect sizes (e.g. Cohen's *d*, Pearson's *r*), indicating how they were calculated

Our web collection on statistics for biologists contains articles on many of the points above.

Software and code

Policy information about availability of computer code

Data collection	The Python processing for PreClinical data pipeline, Pypreclin version 1.0.1, is freely available at https://github.com/neurospin/pypreclin.<br>FMRIB Software Library (FSL) is freely available online (http://www.fmrib.ox.ac.uk/fsl/; version accessed February 4, 2018).
Data analysis	Gradients were computed using the freely available BrainSpace toolbox (http://github.com/MICA-MNI/BrainSpace) for MATLAB (version 2019a), and further MATLAB code for gradient dispersion calculation is freely available from Huang et al (2023) at https://doi.org/10.5281/zenodo.6955280. Third-party MATLAB code to quantify hierarchical integration and segregation from 58 is freely available at https://github.com/TobousRong/Hierarchical-module-analysis. Third-party Python software (version 3.8 was used) for Dominance Analysis is freely available at https://github.com/dominance-analysis/dominance-analysis. Third-party code for Bayes Factor Functions (package: BFF) in R (version 4.3.1) is freely available at https://cran.r-project.org/web/packages/BFF/index.html.

For manuscripts utilizing custom algorithms or software that are central to the research but not yet described in published literature, software must be made available to editors and reviewers. We strongly encourage code deposition in a community repository (e.g. GitHub). See the Nature Portfolio guidelines for submitting code & software for further information.

Data

Policy information about availability of data

All manuscripts must include a data availability statement. This statement should provide the following information, where applicable:

- Accession codes, unique identifiers, or web links for publicly available datasets
- A description of any restrictions on data availability
- For clinical datasets or third party data, please ensure that the statement adheres to our policy

Source data are provided with this manuscript. For the Multi-anaesthesia dataset, raw data are available for access from author B.J. through academic collaboration. For the DBS dataset, raw data are available for access from author B.J. through academic collaboration.

Research involving human participants, their data, or biological material

Policy information about studies with human participants or human data. See also policy information about sex, gender (identity/presentation), and sexual orientation and race, ethnicity and racism.

Reporting on sex and gender	No human data.
Reporting on race, ethnicity, or other socially relevant groupings	No human data.
Population characteristics	No human data.
Recruitment	No human data.
Ethics oversight	No human data.

Note that full information on the approval of the study protocol must also be provided in the manuscript.

Field-specific reporting

Please select the one below that is the best fit for your research. If you are not sure, read the appropriate sections before making your selection.

☒ Life sciences ☐ Behavioural & social sciences ☐ Ecological, evolutionary & environmental sciences

For a reference copy of the document with all sections, see nature.com/documents/nr-reporting-summary-flat.pdf

Life sciences study design

All studies must disclose on these points even when the disclosure is negative.

Sample size	Five rhesus macaques were included for analyses (Macaca mulatta, one male, monkey J, and four females, monkey A, K, Ki, and R, 5-8 kg, 8-12 yr of age). For the DBS dataset, details were provided in (Tasserie et al., 2022). Five male rhesus macaques (Macaca mulatta, 9 to 17 years and 7.5 to 9.1 kg) were included, three for the awake (non-DBS) experiments (monkeys B, J, and Y) and two for the DBS experiments (monkeys N and T). No statistical methods were used to determine sample size, but these sample sizes are in line with similar studies in the field, due to the ethical and practical challenges of neuroscience research with nonhuman primates.
Data exclusions	See "Noise and artifact removal" section below, for quality control criteria used to exclude individual trials from analysis. Exclusion was done prior to analysis.
Replication	Our results using deep propofol anaesthesia were successfully replicated in the same animals using two different anaesthetics, sevoflurane and ketamine (multi-anaesthesia dataset), and they were also successfully replicated in an independent propofol anaesthesia dataset (as part of the DBS dataset analysis).
Randomization	Three monkeys were used for each condition: awake state (monkeys A, K, and J), ketamine (monkeys K, R and Ki), propofol (monkeys K, R, and J), sevoflurane (monkeys Ki, R, and J). The design involves taking multiple samples from the same animals in different conditions, and we controlled for this with linear mixed effects modelling.<br>For the multi-anaesthesia dataset, the acquisitions were performed over 5 years. Whenever possible, animals were scanned in different anesthesia (propofol, sevoflurane, ketamine) conditions.<br>Monkey R: moderate propofol, deep propofol, moderate sevoflurane, deep sevoflurane, ketamine<br>Monkey K: moderate propofol, deep propofol, ketamine<br>Monkey K could not be scanned under moderate sevoflurane and deep sevoflurane anesthesia, because this monkey had health issues not related with this study.<br>Monkey Ki: moderate sevoflurane, deep sevoflurane, ketamine<br>Monkey J: moderate propofol, deep propofol, moderate sevoflurane, deep sevoflurane<br>For the awake condition, monkeys need to have a headpost an be trained for awake fMRI studies. 2 monkeys of the multi-anaesthesia dataset could be scanned in the awake condition. Monkey K: and monkey J. The third monkey of this group was monkey A.

Regarding the DBS dataset, animals and experimental conditions (different location and stimulation for resting state) were randomly chosen using a Matlab function.

Blinding	None: anaesthesia and recovery of responsiveness need to be assessed behaviourally so blinding is not possible.

Reporting for specific materials, systems and methods

We require information from authors about some types of materials, experimental systems and methods used in many studies. Here, indicate whether each material, system or method listed is relevant to your study. If you are not sure if a list item applies to your research, read the appropriate section before selecting a response.

Materials & experimental systems

n/a	Involved in the study
☒	☐ Antibodies
☒	☐ Eukaryotic cell lines
☒	☐ Palaeontology and archaeology
☐	☒ Animals and other organisms
☒	☐ Clinical data
☒	☐ Dual use research of concern
☒	☐ Plants

Methods

n/a	Involved in the study
☒	☐ ChIP-seq
☒	☐ Flow cytometry
☐	☒ MRI-based neuroimaging

Animals and other research organisms

Policy information about studies involving animals; ARRIVE guidelines recommended for reporting animal research, and Sex and Gender in Research

Laboratory animals	Multi-anaesthesia dataset: Macaca mulatta (one male and four females, 8-12 yr of age).<br>DBS dataset: Macaca mulatta (five males, 9 to 17 years).
Wild animals	The study did not involve Wild animals
Reporting on sex	For the anaesthesia dataset, five rhesus macaques were included for analyses (Macaca mulatta, one male, monkey J, and four females, monkey A, K, Ki, and R, 5-8 kg, 8-12 yr of age).<br><br>For the DBS dataset, five male rhesus macaques (Macaca mulatta, 9 to 17 years and 7.5 to 9.1 kg) were included, three for the awake (non-DBS) experiments (monkeys B, J, and Y) and two for the DBS experiments (monkeys N and T).<br><br>Sex was not considered in this study. Because of the small sample sizes, the sex balance per group could not be secured.<br><br>Only males were included in the DBS dataset in order to avoid the menstrual cycle and hormone variations.
Field-collected samples	The study did not used Field-collected samples
Ethics oversight	All procedures are in agreement with the European Convention for the Protection of Vertebrate Animals used for Experimental and Other Scientific Purposes (Directive 2010/63/EU) and the National Institutes of Health’s Guide for the Care and Use of Laboratory Animals. Animal studies were approved by the institutional Ethical Committee (Commissariat à l'Energie atomique et aux Énergies alternatives; Fontenay aux Roses, France; protocols CETEA \#10-003 and 12-086). All procedures are in agreement with 2010/63/UE, 86-406, 12-086 and 16-040.

Note that full information on the approval of the study protocol must also be provided in the manuscript.

Magnetic resonance imaging

Experimental design

Design type	Resting state
Design specifications	For the anaesthesia dataset, a total of 157 functional magnetic imaging runs were acquired (Uhrig et al., 2018): Awake, 31 runs (monkey A, 4 runs; monkey J, 18 runs; monkey K, 9 runs), Ketamine, 25 runs (monkey K, 8 runs; monkey Ki, 7 runs; monkey R, 10 runs), Light Propofol, 25 runs (monkey J, 2 runs; monkey K, 10 runs; monkey R, 12 runs), Deep Propofol, 31 runs (monkey J, 9 runs; monkey K, 10 runs; monkey R, 12 runs), Light Sevoflurane, 25 runs (monkey J, 5 runs; monkey Ki, 10 runs; monkey R, 10 runs), Deep Sevoflurane anaesthesia, 20 runs (monkey J, 2 runs; monkey Ki, 8 runs; monkey R, 11 runs). For details, check the supplementary tables for (Barttfeld et al., 2015; Uhrig et al., 2018; Signorelli et al., 2021) (http://links.ww.com/ALN/B756).

For the DBS dataset, a total of 199 Resting State functional MRI runs were acquired: Awake 47 runs (monkey B: 18 runs; monkey J: 13 runs; monkey Y: 16 runs), anaesthesia (DBS-off) 38 runs (monkey N: 16 runs,; monkey T: 22 runs), low amplitude centro-median thalamic DBS 36 runs (monkey N: 18 runs; monkey T: 18 runs), low amplitude ventro-lateral thalamic DBS 20 runs (monkey T), high amplitude centro-median thalamic DBS 38 runs (monkey N: 17 runs; monkey T: 21 runs), and high amplitude ventro-lateral thalamic DBS 20 runs (monkey T: 20 runs).

Behavioral performance measures

We used a preclinical behavioural scale adapted from Uhrig et al66 to assess the arousal levels of the monkeys. This scale, based on the Human Observers Assessment of Alertness and Sedation Scale113 and previously utilised in non-human primate (NHP) research114, was used consistently across all experimental conditions, in both datasets.
The assessment encompassed six criteria as follows:

- exploration of the surrounding world, from 0 to 2:
0 = total absence,
1 = small search of external clue,
2 = total investigation of the environment (such as head orientation to a sound);
- spontaneous movements, from 0 to 2:
0 = total absence,
1 = small torso and/or limb movement,
2= large torso and/or limb movement
 - shaking / prodding, from 0 to 2:
0= total absence,
1= small body movement,
2 = large body movement;
- toe pinch, from 0 to 2:
0 = total absence,
1 = small reflex (weak body movement or eye blinking or cardiac rate change),
2 = clear reaction (strong body movement and eye blinking or eye opening and cardiac rate change);
 - eyes opening, from 0 to 2:
0 = total absence,
1 = small blinks or eye movements,
2 = full eye opening;
 - corneal reflex, from 0 to 1:
0 = absent,
1 = present.

Acquisition

Imaging type(s)

Functional

Field strength

3T

Sequence & imaging parameters

For the awake condition, monkeys were implanted with a magnetic resonance compatible head post and trained to sit in the sphinx position in a primate chair (Uhrig, Dehaene and Jarraya, 2014)). For the awake scanning sessions, monkeys sat inside the dark magnetic resonance imaging scanner without any task and the eye position was monitored at 120 Hz (Iscan Inc., USA). The eye-tracking was performed to make sure that the monkeys were awake during the whole scanning session and not sleeping. The eye movements were not regressed out from rfMRI data. For the anesthesia sessions, animals were positioned in a sphinx position, mechanically ventilated, and their physiologic parameters were monitored. No eye-tracking was performed in anesthetic conditions. For the anesthesia dataset, before each scanning session, a contrast agent, monocrystalline iron oxide nanoparticle (Feraheme, AMAG Pharmaceuticals, USA; 10 mg/kg, intravenous), was injected into the monkey's saphenous vein (Vanduffel et al., 2001). Monkeys were scanned at rest on a 3-Tesla horizontal scanner (Siemens Tim Trio, Germany) with a single transmit-receive surface coil customized to monkeys. Each functional scan consisted of gradient-echo planar whole-brain images (repetition time = 2,400 ms; echo time = 20 ms; 1.5-mm3 voxel size; 500 brain volumes per run).

For the DBS dataset, monkeys were scanned at rest on a 3-Tesla horizontal scanner (Siemens, Prisma Fit, Erlanger Germany) with a customized eight-channel phased- array surface coil (KU Leuven, Belgium). The parameters of the functional MRI sequences were: echo planar imaging (EPI), TR = 1250 ms, echo time (TE) = 14.20 ms, 1.25-mm isotropic voxel size and 500 brain volumes per run.

Area of acquisition

Whole brain

Diffusion MRI ☒ Used ☐ Not used

Parameters

Anatomical (structural) connectivity data were derived from the recent macaque connectome of (Shen et al., 2019), which combines diffusion MRI tractography with axonal tract-tracing studies, representing the most complete representation of the macaque connectome available to date. Structural (i.e., anatomical) connectivity data are expressed as a matrix in which the 82 cortical regions of interest are displayed in x-axis and y-axis. Each cell of the matrix represents the strength of the anatomical connection between any pair of cortical areas.

Preprocessing

Preprocessing software

Images were preprocessed using Pypreclin (Python preclinical pipeline) (Tasserie et al., Neuroimage 2020). Functional images were corrected for slice timing and B0 inhomogeneities, reoriented, realigned, resampled (1.0 mm isotropic), masked, coregistered to the MNI macaque brain template (Frey et al., 2011), and smoothed (3.0-mm Gaussian kernel). Anatomical images were corrected for B1 inhomogeneities, normalised to the anatomical MNI macaque brain template, and masked.

Normalization

Functional images were reoriented, realigned, and rigidly coregistered to the anatomical template of the monkey Montreal Neurologic Institute (Montreal, Canada) space with the use of Python programming language and Oxford Centre Functional Magnetic Resonance Imaging of the Brain Software Library software (United Kingdom, http://www.fmrib.ox.ac.uk/fsl/; accessed February 4, 2018) (Uhrig, Dehaene and Jarraya, 2014)).

Normalization template

Data were parcellated according to the Regional Map parcellation (Kötter and Wanke, 2005). This parcellation comprises 82 cortical ROIs (41 per hemisphere; Supplementary Table 4).

Noise and artifact removal

Voxel time series were filtered with low-pass (0.05-Hz cutoff) and high-pass (0.0025-Hz cutoff) filters and a zero-phase fast-Fourier notch filter (0.03 Hz) to remove an artifactual pure frequency present in all the data (Barttfeld et al., 2015; Uhrig et al., 2018). Furthermore, an extra quality control (QC) cleaning procedure was performed to ensure the quality of the data after time-series extraction72. This quality control procedure is based on trial-by-trial visual inspection by an expert neuroimager (C.M.S.), and it is the same as was previously implemented in Signorelli et al72. Its adoption ensures that we employ consistent criteria across our two datasets, by adopting the more stringent of the two. We plotted the time series of each region, as well as the static functional connectivity matrix (FC), the dynamic connectivity (dFC) and a Fourier analysis to detect unconventional spikes of activity. For each dataset, visual inspection was first used to become familiar with the characteristics of the entire dataset: how the amplitude spectrum, timeseries, FC and dynamic FC look. Subsequently, each trial was inspected again with particular focus on two main types of potential artefacts. The first one may correspond to issues with the acquisition and is given by stereotyped sinusoidal oscillatory patterns without variation. The second one may correspond to a head or other movement not corrected properly by our preprocessing procedure. This last artefact can be sometimes recognized by bursts or peaks of activity. Sinusoidal activity generates artificially high functional correlation and peak of frequencies in the Amplitude spectrum plot. Uncorrected movements generate peaks of activity with high functional correlation and sections of high functional correlations in the dynamical FC matrix. If we observed any of these anomalies we rejected the trial, opting to adopt a conservative policy. See Figures S17-S19 for examples of artifact-free and rejected trials.

As a result, for the Multi-Anaesthesia data set a total of 119 runs are analysed in subsequent sections (the same as used in Signorelli et al. 72): awake state 24 runs, ketamine anaesthesia 22 runs, light propofol anaesthesia 21 runs, deep propofol anaesthesia 23 runs, light sevoflurane anaesthesia 18 runs, deep sevoflurane anaesthesia 11 runs. For the DBS data set, a total of 156 runs are analysed in subsequent sections: awake state 36 runs, Off condition (propofol anaesthesia without stimulation) 28 runs, low-amplitude CT stimulation 31 runs, low-amplitude VT stimulation 18 runs, high-amplitude CT stimulation 25 runs, high-amplitude VT stimulation 18 runs.

Volume censoring

Not applied.

Statistical modeling & inference

Model type and settings

In figure 1,. Box plots indicate the median and interquartile range of the distribution. * $p < 0.05$; ** $p < 0.01$; *** $p < 0.001$ (FDR-corrected), compared against Awake condition; see Figure S1 for comparisons between all pairs; see Figure S2 for gradient 2 results).

In figure 4, Box plots indicate the median and interquartile range of the distribution. * $p < 0.05$; ** $p < 0.01$; *** $p < 0.001$ (FDR-corrected), compared against Awake condition; * $p < 0.05$; ** $p < 0.01$; *** $p < 0.001$ (blue asterisks in the article) (FDR-corrected), compared against CT high condition; see Figure S3 for comparisons between all pairs, and Figure S4 for hierarchical segregation).

I figure 7, Box plots indicate the median and interquartile range of the distribution. * $p < 0.05$; ** $p < 0.01$; *** $p < 0.001$ (FDR-corrected), compared against Awake condition; * $p < 0.05$; ** $p < 0.01$; *** $p < 0.001$ (FDR-corrected), compared against CT high condition; see Figure S5 for comparisons between all pairs

Effect(s) tested

Figure 8 show the magnitude (absolute value) of the effect size obtained when comparing each state of perturbed consciousness against wakefulness for the multi-anaesthesia data set and the DBS dataset.

Figures S1, S3 and S5 show effect sizes for significant changes across states of consciousness. Matrices show the effect sizes for each statistical contrast between conditions of the multi-anaesthesia and DBS datasets; positive (negative) values indicate that the condition in the row is greater (lower) than the condition in the column. White entries indicate that the difference is not statistically significant.

Specify type of analysis: [x] Whole brain [ ] ROI-based [ ] Both

Statistic type for inference

(See Eklund et al. 2016)

We used Whole brain statistical analysis techniques using the Cocomac Parcellation with 82 cortical regions.

Correction

FDR corrections are used in this article in figures 1,2,4,and 7 in the main text and S2 and S4 in the supplementary material.

Models & analysis

n/a	Involved in the study
[ ]	[x] Functional and/or effective connectivity
[ ]	[x] Graph analysis
[ ]	[x] Multivariate modeling or predictive analysis

Functional and/or effective connectivity

For the Functional Gradient Mapping, we calculate the functional connectivity matrix (FC) as the Pearson correlation between each pair of regional fMRI signals per scan per condition. Following previous work, each matrix was z-transformed and thresholded row-wise to achieve 90% sparsity, retaining only the strongest connections in each row. The cosine similarity matrix was calculated on the thresholded z-matrix to generate a similarity matrix reflecting the similarity in whole-brain connectivity patterns between vertices.

Graph analysis

We used Diffusion Map Embedding, that exploits the properties of the graph Laplacian to model the diffusion process. The relative influence of density of sampling points on the manifold is controlled by an additional parameter in the range of 0 to 1, which for diffusion map embedding is set to 0.5 to provide a balance between local and global contributions to the embedding space estimation. The high-dimensional similarity matrix is treated as a graph, with "connections" (entries of the similarity matrix) reflecting the similarity between the regional patterns of FC.
Following the method developed by Atasoy and colleagues (Atasoy, Donnelly and Pearson, 2016), we compute the symmetric graph Laplacian   on the matrix   that represents the macaque structural connectome. Thereafter, we estimate the connectome Laplacian (discrete counterpart of the Laplace operator   applied to the network of the macaque structural brain connectivity) and used its eigenvectors to decompose functional activation.

Multivariate modeling and predictive analysis

We compute gradients using the dimensionality reduction technique known as diffusion map embedding. The technique estimates a low-dimensional set of embedding components (gradients); in this low-dimensional space, proximity reflects similarity of the patterns of FC: regions with similar FC patterns (which are strongly connected in the network) are placed close to each other, and regions with low similarity are placed far apart. In this way, each gradient represents one dimension of covariance in the inter-regional similarity between FC patterns, with a small number of gradients capturing most of the dimensions of inter-regional similarity, which can then be visualised by a low-dimensional scatter plot (Figure 4).

Dominance Analysis: Unlike other methods of assessing predictor importance, such as methods based on regression coefficients or univariate correlations, dominance analysis accounts for predictor-predictor interactions and its interpretation is straightforward. Specifically, dominance analysis seeks to determine the relative contribution (“dominance”) of each independent variable to the overall fit (adjusted R2) of a multiple linear regression model64,65. This is done by fitting the same regression model on every combination of predictors (2p − 1 submodels for a model with p being the predictors). Total dominance is defined as the average of the relative increase in R2 when adding a single predictor of interest to a submodel, across all 2p − 1 submodels. The sum of the dominance of all input variables is equal to the total adjusted R2 of the complete model, from which a percentage of relative importance is obtained by partitioning the total variance accounted for by each predictor.

Here, we use as regressors the data pertaining to our three markers of interest: range of the principal gradient, hierarchical integration, and harmonic energy. For this analysis, we combine data across the Multi-Anaesthesia and DBS datasets. As target variable, we use the arousal score corresponding to each condition, on a scale from 0 to 1166. To ensure that data could be combined across the DBS and Multi-Anaesthesia datasets, values of each marker were normalised between 0 and 1 separately within each dataset (by subtracting the minimum value observed within the dataset, and then dividing by the maximum) before aggregating the two datasets.

In addition to using dominance analysis for multiple regression, it is also possible to use it for multivariate classification. To do so, we dichotomised the arousal scores: either using a cut-off of 9, to combine together wakefulness and the effects of high-amplitude CT stimulation, against everything else (corresponding to the difference between high arousal and low or no arousal); or using a cut-off value of 3 instead, thereby separating deep anaesthesia and VT stimulation, from wakefulness, CT stimulation, and light anaesthesia (i.e., no arousal versus any level of arousal).
